# Skylight Polarization Pattern Simulator Based on a Virtual-Real-Fusion Framework for Urban Bionic Polarization Navigation

**DOI:** 10.3390/s23156906

**Published:** 2023-08-03

**Authors:** Qianhui Li, Liquan Dong, Yao Hu, Qun Hao, Jiahang Lv, Jie Cao, Yang Cheng

**Affiliations:** Key Laboratory for Precision Optoelectronic Measurement Instrument and Technology, School of Optics and Photonics, Beijing Institute of Technology, Beijing 100081, China

**Keywords:** bionic polarization navigation, skylight polarization pattern, data preparation, machine learning, atmospheric model, polarimeter

## Abstract

In a data-driven context, bionic polarization navigation requires a mass of skylight polarization pattern data with diversity, complete ground truth, and scene information. However, acquiring such data in urban environments, where bionic polarization navigation is widely utilized, remains challenging. In this paper, we proposed a virtual-real-fusion framework of the skylight polarization pattern simulator and provided a data preparation method complementing the existing pure simulation or measurement method. The framework consists of a virtual part simulating the ground truth of skylight polarization pattern, a real part measuring scene information, and a fusion part fusing information of the first two parts according to the imaging projection relationship. To illustrate the framework, we constructed a simulator instance adapted to the urban environment and clear weather and verified it in 174 urban scenes. The results showed that the simulator can provide a mass of diverse urban skylight polarization pattern data with scene information and complete ground truth based on a few practical measurements. Moreover, we released a dataset based on the results and opened our code to facilitate researchers preparing and adapting their datasets to their research targets.

## 1. Introduction

In the walk of extensive urban deployment of unmanned platforms, urban environments have put forward even greater challenges to conventional navigation and positioning technologies. Long-distance working of an inertial navigation system can result in accumulating errors [1]. Dense electromagnetic interference in cities can mislead geomagnetic navigation systems [2]. High-rise urban buildings can block satellite signals and hinder the global satellite navigation system from functioning. The bionic polarization navigation intrigues researchers by virtue of its preferable resistance to both accumulated errors and electromagnetic interferences [3,4,5]. The bionic polarization navigation exploits the skylight polarization pattern across the celestial dome to identify directions [6,7]. The sunlight keeps unpolarized until it enters the terrestrial atmosphere, where particles scatter the sunlight and change its polarization state regularly so that the polarization characteristic of the skylight manifests a typical pattern across the whole celestial dome [8]. Since the pattern is closely related to the relative location of the sun and the observing site, it contains abundant directional information and offers a reliable information source for navigation. 

In today’s data-driven research context, more and more researchers are trying to promote bionic polarization navigation with machine learning methods [9,10,11,12,13], which has created requirements for skylight polarization pattern data in terms of volume and content. Firstly, the volume of the dataset is an important factor for machine learning’s performance. Experiments have proved that as the size and diversity of data increase, the accuracy of the machine learning algorithms improves very much [14]. Hence a mass of diverse skylight polarization patterns needs to be collected as materials for the machine’s learning. Secondly, the content of the dataset is also significant. The data are supposed to be as close to the real application scenario as possible [15] so that the learning results are more likely to reveal the physical scattering essence of the specific scenario. Data close to the real application scenario improve the generalization ability of the network model in applications as well. The complete ground truth of the skylight polarization pattern is also necessary, especially for supervised learning methods. This is because the data collected in applications are generally defective. The neural network can be trained to make correct predictions based on defective data, but the ground truth of the defects is needed as a reference target, and the training is supervised by bias between the machine’s predictions and the conclusion based on ground truth [16]. 

In order to acquire eligible skylight polarization pattern data, two approaches have been developed. However, some problems still exist when these approaches are applied in urban environments where navigation is widely demanded. Acquiring a mass of skylight polarization pattern data with diversity, scenario information, and complete ground truth remains a challenge in urban environments.

On the one hand, many researchers are trying to acquire skylight polarization pattern data through practical measurements. Various imaging polarimeters, including the division-of-time polarimeter [17,18,19], the division-of-channel polarimeter [12,20], and the division-of-focal-plane (DoFP) polarimeter [21,22], have been established. Although the widely used DoFP polarimeter nowadays is much more compact and convenient compared to the others [23], practically collecting skylight polarization patterns to build a dataset is still a labor-intensive and time-consuming task. In order to obtain a large number of skylight polarization pattern data with great diversity, multiple measurements should be conducted at different places. To obtain a specific skylight polarization pattern under an urban environment, one has to wait until the sun moves to the corresponding position. In addition, the sky scene in urban environments is always partially obscured by many landscapes including buildings, street lights, and trees. The skylight polarization pattern in the obscured regions is inaccessible. Some researchers are trying to overcome obscurations by inpainting the obscured region in the skylight polarization pattern [24]. However, it is difficult to access the ground truth of the skylight polarization pattern in the obscured regions through practical measurement. Because of the lack of ground truth, such a dataset cannot provide effective supervision for the inpainting process. 

On the other hand, some researchers are also trying to construct skylight polarization pattern datasets through numerical simulation methods. To precisely describe the skylight polarization pattern, various atmospheric polarization models that adapt to different atmospheric conditions, such as the Rayleigh single scattering model [8], the Berry model [25], and the Hošek–Wilkie model [26], have been proposed. On this basis, Wang et al. [11,27] numerically generated a skylight polarization pattern dataset based on the Berry model and applied it to a navigation neural network. Liang et al. [10,28,29] numerically simulated the skylight polarization pattern during the whole process from generating to imaging and released a polarized skylight navigation simulation dataset. Although Liang et al. creatively considered the acquisition process of the skylight polarization pattern and added artificial noise to approximate the real measurement, the existing simulation methods stayed within the bounds of the numerical simulation. As we mentioned, the urban sky scene is inevitably obscured by landscapes. Numerical simulation methods cannot reflect the real scene information of the urban environments, which can lead to a dataset without the sample features required for practical application and generalization.

To address the issue, we propose a universal virtual-real-fusion framework to prepare skylight polarization pattern data, which complements the defects of both methods of practical measurement and numerical simulation. The framework consists of three parts: the virtual part based on the numerical simulation, the real part based on the practical measurement, and the fusion part based on calibration. In the following sections, we elaborate on the framework, taking an instance of a skylight polarization pattern simulator established and adapted to the urban landscape and clear weather. The structure of the simulator is introduced in detail in Section 2. In Section 3, experiments are conducted and a dataset is constructed based on experimental results. The simulator is verified from the perspective of the dataset’s volume and content. In Section 4, we discuss the probable prospect of the framework. Because the specific interaction between the skylight and landscapes is too intricate to predict mathematically, the current fusion stays at the contour level. This limitation is also discussed in Section 4 and it remains to be worked out in future studies. Moreover, to facilitate researchers to prepare skylight polarization patterns adapted to their scenarios and devices, we disclose the source code of the simulator on GitHub. 

## 2. Urban Skylight Polarization Simulator

**Figure 1:**
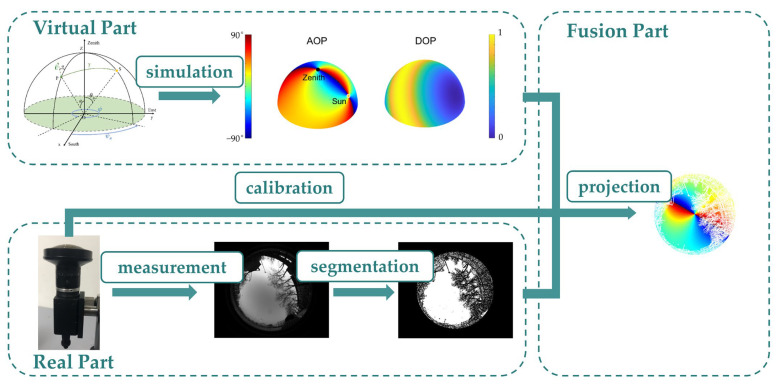
Flowchart of the virtual-real-fusion framework for the urban skylight polarization pattern simulator.

The flowchart of the virtual-real-fusion framework is shown in Figure 1. Herein, the virtual part is based on an atmospheric polarization model, which simulates the expected skylight polarization pattern as a three-dimensional (3D) hemisphere. The pattern is characterized by the angle of polarization (AOP) and the degree of polarization (DOP). The real part is based on an imaging polarimeter, which captures the scene information in the form of two-dimensional (2D) images. The fusion part associates the 3D model with the 2D image, i.e., the virtual skylight polarization pattern with the real scenario information, through the process of calibration and projection. In order to elaborate on the virtual-real-fusion framework, we constructed a skylight polarization pattern simulator adapted to the urban landscape and clear weather as an instance. 

### 2.1. Virtual Part

To acquire the skylight polarization pattern of the whole celestial dome with diversity and complete ground truth in a time- and labor-saving way, a virtual part was devised in our simulator. This part models the celestial dome as a 3D hemisphere and numerically simulates the skylight polarization pattern as the distribution of the angle of polarization (AOP) and the degree of polarization (DOP) across the hemisphere. 

**Figure 2:**
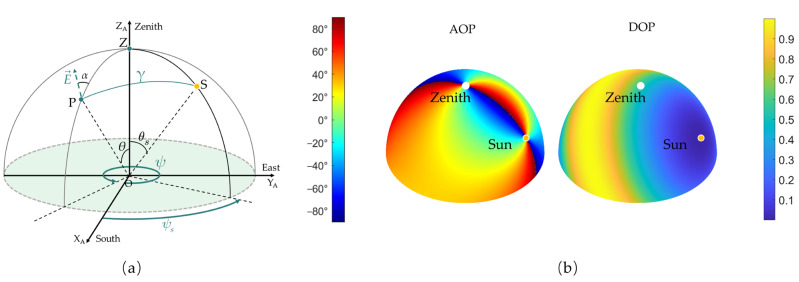
(**a**) Atmospheric coordinate system of the skylight polarization pattern of the whole celestial dome. (**b**) The ideal skylight polarization pattern (*w* = 1) simulated in a 3D atmospheric model when the coordinate of the sun is $\left(\frac{\pi }{3},\frac{\pi }{4}\right)$.

An atmospheric coordinate system OX_A_Y_A_Z_A_ was established as shown in Figure 2a. The origin of the coordinate system is at the observing position point O on the Earth. The X_A_-axis points geographical south, the Y_A_-axis points geographical east, and the Z_A_-axis points in the direction of the zenith. Herein, the celestial dome observed at the observing position on the earth is modeled as a hemisphere of normalized radius 1, and an arbitrary point on the hemisphere can be characterized by the zenith angle θ and the azimuth angle ψ. The $S(\theta_{s},\psi_{s})$ and P(θ,ψ) on the hemisphere represent the position of the sun and the scattering particle, respectively. Point Z represents the zenith. 

The main composition of the atmosphere in clear weather is gas molecules, such as nitrogen and oxygen accounting for about 78% and 21%, respectively. Their scale is much smaller than the wavelength of visible light and thus falls into the range of the Rayleigh scattering. Therefore, in this simulator, we selected the Rayleigh scattering model to simulate the skylight polarization pattern adapted to clear weather. According to Rayleigh scattering theory, the polarization property of the scattered light is mainly determined by the scattering angle *γ*, i.e., the angle between the incident and scattered beams. Rayleigh theory predicts the DOP of the scattered beam as Equation (1) [8], where *w* is a weighting that denotes the maximum DOP of the whole sky and equals 1 for an ideal Rayleigh single scattering model. The polarized electric field vector ($\vec{E}$) of the scattered beam keeps perpendicular to the scattering plane defined by the incident and scattered beams.

(1) $$\mathrm{DOP}=w\frac{1-\cos^{2}\gamma }{1+\cos^{2}\gamma }.$$

In our coordinate system, because the sunlight reaching the earth’s atmosphere is parallel, the sunlight beam reaching the particle at P(θ,ψ) is regarded as going along the SO direction. The scattered beam by P reaches the observing point O along the PO direction. Hence, the scattering angle *γ* equals the angular distance between the sun $S(\theta_{s},\psi_{s})$ and the particle P(θ,ψ) and the scattering plane turns out to be parallel to the plane OPS. The equation for *γ* is derived from the law of cosines to the spherical triangle as follows:

(2) $$\cos\gamma =\cos\theta_{s}\cos\theta +\sin\theta_{s}\sin\theta \cos(\psi_{s}-\psi ).$$

The direction of the polarized electric field vector ($\vec{E}$) is represented by the angle *α* with respect to a reference plane OPZ. α is defined as the AOP. The equation for *α* viewed by the camera is derived as follows:

(3) $$\tan\alpha =\frac{\cos\theta_{s}\sin\theta -\sin\theta_{s}\cos\theta \cos(\psi -\psi_{s})}{\sin(\psi_{s}-\psi )\sin\theta_{s}}.$$

As a result, the skylight polarization characteristics at the position of P can be calculated as Equation (4). The skylight polarization pattern is obtained by performing the same calculation for each point on the hemisphere. Typically, the ideal skylight polarization pattern on the whole celestial dome is simulated in Figure 2b when the sun is settled at the position of, for example, $\left(\frac{\pi }{3},\frac{\pi }{4}\right)$ and *w* equals 1. The scattered light remains unpolarized when *γ* equals 0°, but is completely polarized when *γ* equals 90°. When P is at a position with another *γ* value, the scattered light is partially polarized light.

(4) $$\left\{\begin{array}{c} \mathrm{DOP}=w\frac{1-{\left(\cos\theta_{s}\cos\theta +\sin\theta_{s}\sin\theta \cos(\psi_{s}-\psi )\right)}^{2}}{1+{\left(\cos\theta_{s}\cos\theta +\sin\theta_{s}\sin\theta \cos(\psi_{s}-\psi )\right)}^{2}}\\ \mathrm{AOP}=\arctan(\frac{\cos\theta_{s}\sin\theta -\sin\theta_{s}\cos\theta \cos(\psi -\psi_{s})}{\sin(\psi_{s}-\psi )\sin\theta_{s}}) \end{array}\right..$$

In the virtual part of the simulator, we built the celestial dome as a 3D hemisphere and numerically simulated the skylight polarization pattern in the clear weather based on the Rayleigh scattering model. As a result, through the virtual part of the simulator, the complete ground truth across the whole celestial dome was obtained, which can support supervised learning methods in the data-driven application. Moreover, a mass of skylight polarization pattern data with diversity can be easily acquired in a short time, and arbitrary patterns can be obtained simply by changing the solar coordinates. 

### 2.2. Real Part

As reviewed in Section 1, the urban sky scene is inevitably obscured by landscapes including buildings, street lights, and trees. Although the virtual part of the simulator can generate the skylight polarization pattern with ground truth in a time- and labor-saving way, it cannot reflect the real landscape information of the urban environments, leading to a lack of sample features required for the practical application and generalization. In order to introduce the urban landscape information into the skylight polarization pattern and make the dataset closer to the application scenario, we constructed the real part of the simulator based on a division-of-focal-plane (DoFP) polarimeter. The polarimeter can capture polarized images of the urban sky scene, and the corresponding skylight polarization pattern can be calculated in the form 2D matrix. With the help of the measured skylight polarization pattern, the landscape and sky regions can also be segmented. 

**Figure 3:**
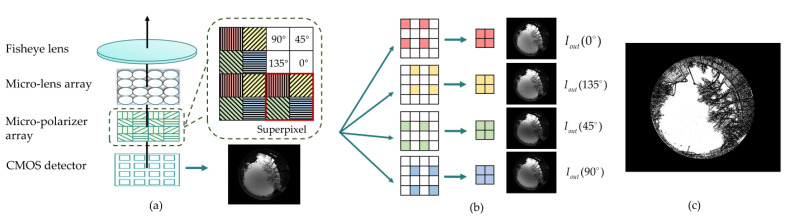
(**a**) The typical internal structure of the full-sky polarization imaging polarimeter [24]. The micro-polarizers with parallel wire grids along 90°, 45°, 135°, and 0° are represented by red, yellow, green, and blue, respectively. (**b**) Schematic of the pixel segmentation process [24]. As displayed in (**a**), the four pixels within a superpixel respectively capture light intensities that linearly polarized with electric fields along 0°, 135°, 45° and 90°. According to this arrangement of the micro-polarizer array, pixels in the images can be split and recombined to form four polarization images which are represented by $I_{out}(0^{\circ })$, $I_{out}(135^{\circ })$, $I_{out}(45^{\circ })$, and $I_{out}(90^{\circ })$. (**c**) Schematic of the *MASK* matrix.

The process of the real part is illustrated in the case of the wide-angle DoFP imaging polarimeter [21,24]. The imaging polarimeter consists of a fisheye objective and a DoFP polarization camera, and the typical internal structure of the polarimeter is abstractly shown in Figure 3a [24]. The fisheye objective placed at the top offers a wide field of view (FOV) to collect the real landscape information of urban sky scenes. From the bottom up, the complementary metal oxide semiconductor (CMOS) image detector is successively covered by a micro-polarizer array and a micro-lens array. The micro-polarizer array assures that the transmitted wave is linearly polarized with an electric field perpendicular to the wires. The micro-lens array reduces the crosstalk of polarized angles by avoiding the beam being incorrectly detected by the wrong pixel. Hence, four polarization images $I_{out}(0^{\circ })$, $I_{out}(135^{\circ })$, $I_{out}(45^{\circ })$, and $I_{out}(90^{\circ })$ can be captured with a single shooting and segmentation. According to the arrangement of micro-polarizers, the segmentation process is schematically conducted as shown in Figure 3b [24].

Using the four captured polarized images $I_{out}(0^{\circ })$, $I_{out}(135^{\circ })$, $I_{out}(45^{\circ })$, and $I_{out}(90^{\circ })$, the skylight polarization pattern of this sky scene can be calculated. The skylight polarization pattern is generally characterized by the angle of polarization (AOP) and the degree of polarization (DOP) in the form of a 2D matrix, and the formula is shown as Equation (5) [30].

(5) $$\left\{\begin{array}{c} \mathrm{AOP}=\frac{1}{2}\arctan\left(\frac{U}{Q}\right)\\ \mathrm{DOP}=\frac{\sqrt{Q^{2}+U^{2}}}{I} \end{array}\right..$$

where, I, Q, and U represent the three components of the Stokes vector of the beam recorded by CMOS and can be calculated using Equation (6) [31]:

(6) $$\left\{\begin{array}{l} I=I_{out}(0^{\circ })+I_{out}(90^{\circ })\\ Q=I_{out}(0^{\circ })-I_{out}(90^{\circ })\\ U=2\times I_{out}(45^{\circ })-I_{out}(0^{\circ })-I_{out}(0^{\circ }) \end{array}\right..$$

As we reviewed in Section 1, the urban sky scene is always partially obscured by some landscapes that cannot be described by pure numerical simulation, and the real part of our simulator is constructed to capture real landscape information of urban environments. Hence, the landscape and the sky should be distinguished in the acquired skylight polarization pattern. Our previous study found that the sky’s DOP falls within a range, but the landscape’s DOP is often beyond the threshold. Additionally, the landscape manifests as bigger gradients of DOP [24]. With the help of the calculated polarization pattern, the sky region and the landscape region can be distinguished following the criteria described in Equation (7) [24]:

(7) $$MASK(i,j)=\left\{\begin{array}{ccc} 1 & , & {\mathrm{DOP}}_{\min}<\mathrm{DOP}(i,j)<{\mathrm{DOP}}_{\max}\\ & & \mathrm{and}\;gradient(\mathrm{DOP}(i,j))<G_{\max}\;\\ 0 & , & \mathrm{other} \end{array}\right.,$$

where DOP(i,j) and gradient(DOP(i,j)) represent the DOP and DOP’s gradient at (i,j), respectively. ${\mathrm{DOP}}_{\min}$ and ${\mathrm{DOP}}_{\max}$ represent the lower and the upper limitations of DOP, and $G_{\max}$ represents the upper limitation for DOP’s gradient. The ${\mathrm{DOP}}_{\min}$, ${\mathrm{DOP}}_{\max}$, and $G_{\max}$ are hyper-parameters whose optimization has been discussed [24], and they equal 0.02, 0.75, and 0.02, respectively, in this paper. As a result, a 2D *MASK* matrix that has the same size as the polarization pattern is generated as shown in Figure 3c. Herein, the region where the MASK(i,j) equals 1 represents the sky region, and the region where the MASK(i,j) equals 0 represents the landscape region. 

Through the real part of the simulator, the real landscape information in the urban sky scene is collected in the form of a 2D matrix. Based on real information, the skylight polarization pattern data generated from our simulator can be equipped with real sample features and better generalization ability in practical applications. 

### 2.3. Fusion Part

To associate the 3D model with the 2D image, i.e., the virtual skylight polarization pattern with the real scene information, the fusion part of the simulator was constructed. The imaging projection relationship between a point in the 3D virtual model and its projection in the measured 2D image is the fusion bridge between the virtual skylight polarization pattern and the real scene information. In this part, we proposed the imaging projection relationship of the virtual-real-fusion framework and fused the real scene information and the virtual skylight polarization pattern according to the relationship.

**Figure 4:**
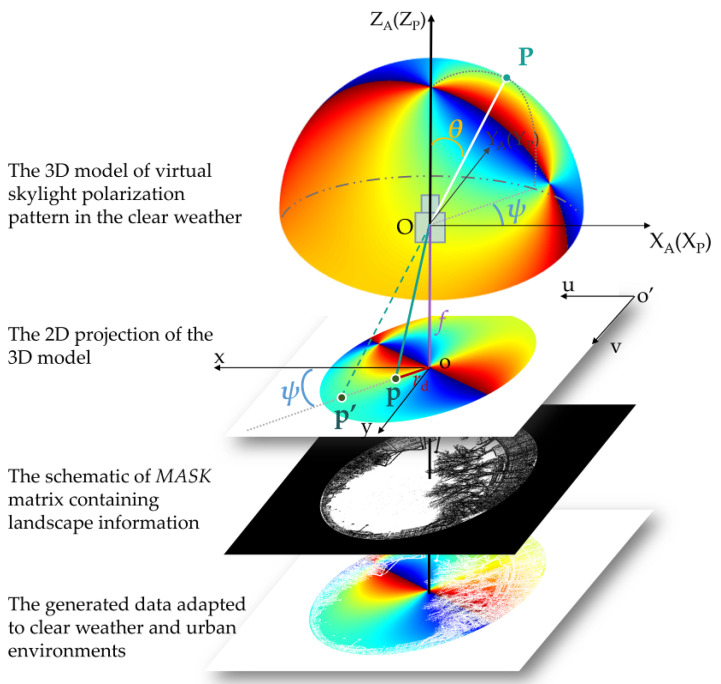
The projection relationship of the virtual-fusion skylight polarization simulator. P is a point in the 3D atmosphere model. From the top down, the 2D images under the 3D model are the 2D projection of the virtual skylight polarization pattern, the schematic of the *MASK* matrix containing landscape information, and the urban skylight polarization pattern. In the projection image of the virtual skylight polarization pattern, the image point of P is point p, whereas it would be point p’ by a pinhole model.

As shown in Figure 4, the projection between the virtual part and the real part involves four coordinate systems: the virtual atmospheric coordinate system OX_A_Y_A_Z_A_, the polarimeter coordinate system OX_P_Y_P_Z_P_, the image coordinate system oxy, and the pixel coordinate system o’uv. As introduced in Section 2.1, the ideal skylight polarization pattern is modeled in the virtual atmospheric coordinate system OX_A_Y_A_Z_A_. The origin of the coordinate system is at the observing position point O on the earth. The X_A_-axis points geographical south, the Y_A_-axis points geographical east, and the Z_A_-axis points the direction of the zenith. P is an arbitrary point on the skylight polarization pattern model. Because the radius of the hemisphere is normalized to 1, the position of P can be characterized with the zenith angle θ, and the azimuth angle ψ. 

The fusion comprises four steps. Firstly, the coordinate transformation from the virtual atmospheric coordinate system OX_A_Y_A_Z_A_ to the polarimeter coordinate system OX_P_Y_P_Z_P_ is conducted. The origin of the rectangular coordinate system OX_P_Y_P_Z_P_ is the polarimeter center. The Z_P_-axis is along the optical axis of the polarimeter. Since the polarimeter is the observing device and the optical axis of the polarimeter points to the zenith, the origin point of the coordinate system OX_P_Y_P_Z_P_ coincides with that of the coordinate system OX_A_Y_A_Z_A_ and the Z_P_-axis coincides with the Z_A_-axis. To make it easier to present, we say the two coordinate systems coincide. The point P can also be expressed as P(θ,ψ) in the polarimeter coordinate system. 

Secondly, the projection relationship between the 3D model and the 2D image is established based on the imaging model of the polarimeter. Random point P on the skylight polarization pattern is captured and imaged by the imaging polarimeter. Since as much information about the real scene is expected as is possible, the fisheye lens with a hemispherical FOV of about 180° is used in the polarimeter in Section 2.2. It is impossible to project the hemispherical FOV on a finite image plane by a classical pinhole imaging model. A fisheye imaging model proposed by Kannala [32] is applicative to our polarimeter, and the model is presented as

(8) $$r_{d}=f\theta_{d},$$

(9) $$\theta_{d}=k_{0}\theta +k_{1}\theta^{3}+k_{2}\theta^{5}+k_{3}\theta^{7}+k_{4}\theta^{9}+\ldots .$$

where *θ* is the angle between the principal axis of the camera and the incident ray. f is the focal length and $r_{d}$ is the distance between the image point and the principal point in the image plane. $k_{0}$, $k_{1}$, $k_{2}$, $k_{3}$, $k_{4}$… are constants to represent the distortion of *θ*, where $k_{0}$ generally defaults to 1. The first five terms give enough degrees of freedom for imaging curves [32], so in this paper only $k_{0}$ to $k_{4}$ is used. 

As introduced in Section 2.1, the sunlight is scattered by the particle at the position of P and the scattered beam is observed by the polarimeter at the position of O, so the scattered beam is along the PO direction. For the polarimeter, the angle *θ* between the principal axis and the incident ray PO is nicely the zenith angle *θ* of point P, which makes the projection process very clear to deduce. According to Equations (8) and (9), the P is projected as point p in the image coordinate system oxy with the following coordinates:

(10) $$\left[\begin{array}{c} x_{d}\\ y_{d} \end{array}\right]=\left[\begin{array}{cc} f_{x} & 0\\ 0 & f_{y} \end{array}\right]\left[\begin{array}{c} \theta_{d}\cos\psi \\ \theta_{d}\sin\psi \end{array}\right],\;\mathrm{where}\;\theta_{d}=\theta +k_{1}\theta^{3}+k_{2}\theta^{5}+k_{3}\theta^{7}+k_{4}\theta^{9}.$$

where $f_{x}$ and $f_{y}$ are the focal lengths in the x and y direction, respectively.

Thirdly, to ensure that the projection result has the same pixel scale as the 2D image captured by the polarimeter, the coordinate of p is transformed in the pixel coordinate system o’uv.

(11) $$\left[\begin{array}{c} u\\ v\\ 1 \end{array}\right]=\left[\begin{array}{ccc} f_{x} & 0 & c_{x}\\ 0 & f_{y} & c_{y}\\ 0 & 0 & 1 \end{array}\right]\left[\begin{array}{c} \theta_{d}\cos\psi \\ \theta_{d}\sin\psi \\ 1 \end{array}\right],\;\mathrm{where}\;\theta_{d}=\theta +k_{1}\theta^{3}+k_{2}\theta^{5}+k_{3}\theta^{7}+k_{4}\theta^{9}.$$

where $c_{x}$ and $c_{y}$ are pixel coordinates of the center of the image. 

Finally, according to the above-shown projection process, the skylight polarization pattern is projected as a 2D image, and they conform to the imaging projection relationship. Consequently, the 2D skylight polarization pattern has the same pixel size as the image captured by the polarimeter, and the pixels on the pattern and the image with the same pixel coordinate correspond one to one. According to Section 2.2, the sky region and the landscape region can be filtered with a *MASK* matrix, and the urban skylight polarization pattern with landscape information is generated. 

In the above-shown projection relationship, eight internal constant parameters of the polarimeter including $k_{1}$, $k_{2}$, $k_{3}$, $k_{4}$, $f_{x}$, $f_{y}$, $c_{x}$, and $c_{y}$ are involved. They can be obtained through calibration experiments [32]. 

Through this part, the real part and the virtual part are favorably combined, which complements the defects of both data acquisition methods of practical measurement and numerical simulation. Following this virtual-real-fusion framework, all the acquired data possess both scene information and complete ground truth to be consulted. Scene information equips the data with sample features required for practical application and generalization. Complete ground truth provides a reference target for supervised training. Meanwhile, arbitrary skylight polarization patterns can be simulated based on one practical measurement, ensuring both the data volume and diversity. 

## 3. Experiments and Results

To verify the effect of the virtual-real-fusion framework, we conducted experiments based on the constructed urban skylight polarization pattern simulator. We captured the real scene images under urban environments with the polarimeter in Section 3.1. Then, we calibrated the polarimeter in Section 3.2. Finally, we projected the ideal skylight polarization model into the 2D images captured by the polarimeter and showed the fusion results in Section 3.3.

### 3.1. The Measurement Experiments and Results

We conducted the practical measurement experiment using the hardware system constructed in our previous study [24]. It was based on a full-sky imaging polarimeter consisting of a fisheye lens FE185C057HA-1 from FUJIFILM and a CMOS polarization image detector IMX250MYR from SONY. The focal length of the fisheye lens was 1.8 mm, with the effective angle of FOV equaling 185° × 185° (H × V). The CMOS chip was composed of 2448 × 2048 pixel units, and the size of each pixel unit was 3.45 μm × 3.45 μm. The polarizers covering the CMOS surface had the same pixel size as the CMOS chip.

With the experimental system, we conducted measurement experiments under 174 different urban conditions. The experimental site was at the Beijing Institute of Technology (geographical coordinates: 116°19′15″ E, 39°57′55″ N), and the weather condition was sunny with good air quality. It is worth mentioning that before shooting, the optical axis of the polarimeter was adjusted to point vertically towards the sky using a gradienter. The orientation of the carrier was adjusted with a compass. This was carried out mainly to ensure that the polarimeter coordinate system coincided with the atmospheric coordinate system.

### 3.2. The Calibration Experiments and Results

In order to figure out the imaging model of the polarimeter and establish the projection relation between the 3D model and the 2D images, we conducted calibration experiments in this section.

**Figure 5:**
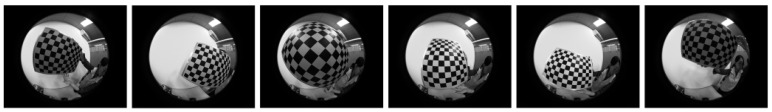
Some pictures of the calibration plane captured by the polarimeter.

According to the projection process introduced in Section 2.3, eight internal constants including $k_{1}$, $k_{2}$, $k_{3}$, $k_{4}$ $f_{x}$, $f_{y}$, $c_{x}$, and $c_{y}$ needed to be calibrated. Firstly, we captured images of the calibration plane using the established imaging polarimeter. The calibration plane was a 12 × 9 square checkboard, and the size of each square was 30 mm. Some captured images are illustrated in Figure 5. 

Secondly, 88 inner corners of the checkerboard in each image were extracted as control points. For each image, we fixed the world coordinate system on the checkerboard [32]. Due to that, the size of the square was known as 30 mm, and the coordinates of a control point in the world coordinate system were known as multiples of 30 mm. 

Thirdly, according to Kannala’s imaging model and calibration method [32], the pixel coordinates of the control points were deduced and the eight parameters were calculated by iteratively minimizing the sum of squared distances between the deduced pixel coordinate and the actual pixel coordinate.

We conducted three calibration experiments in total, and 40, 40, and 37 frames of images were captured, respectively. The results of the calibration experiments are shown in Table 1. The mean values of three experiments were taken as parameters of the polarimeter and used in fusion in Section 3.3.

### 3.3. Fusion Results

Based on above experiments, we realized the fusion of virtual ideal skylight polarization model and the real urban scene images. We projected the 3D skylight polarization model into 2D image captured in Section 3.1 according to the imaging parameters estimated in Section 3.2. 

**Figure 6:**
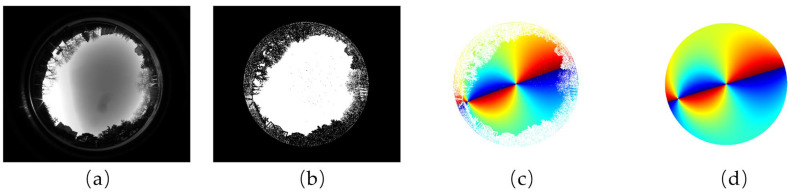
Fusion results of the simulator. (**a**) The image of the urban sky scene, (**b**) the schematic of the *MASK* matrix of the urban scene, (**c**) the urban skylight polarization pattern acquired by our simulator when the position of the sun is set at $\left(\frac{5}{12}\pi ,-\frac{8}{9}\pi \right)$, and (**d**) the ground truth of the skylight polarization pattern.

The fusion results are illustrated with an example shown in Figure 6. From left to right, Figure 6a shows an image of the urban sky scene captured by the polarimeter. By virtue of the fisheye lens in the polarimeter, the image has a wide FOV to capture not only the sky region but also the landscape information. Figure 6b shows the segmentation effect of the *MASK* matrix. The white region where the MASK(i,j) equals 1 represents the sky region, and the black region where the MASK(i,j) equals 0 represents the landscape region. The sky region and the landscape region in Figure 6a are nicely distinguished by the *MASK* matrix. By setting the coordinate of the sun as $\left(\frac{5}{12}\pi ,-\frac{8}{9}\pi \right)$ t and projecting the 3D model to the 2D plane, we obtained the urban skylight polarization pattern corresponding to the scene in Figure 6c. It provides not only an ideal skylight polarization pattern but also real urban scene information. Figure 6d shows the complete ground truth of the skylight polarization pattern in Figure 6c. That is to say, with this simulator, the obscured regions of skylight polarization patterns turn out to be known, and the ground truth can provide a reference to related research and applications, such as pattern inpainting and supervised learning.

**Figure 7:**
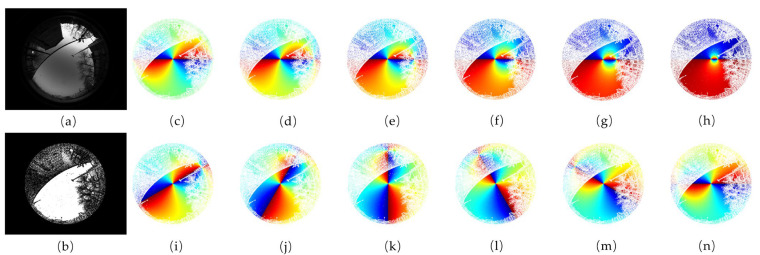
The diversity of skylight polarization patterns demonstrated by fusion results under the same urban environment when the sun is at a different position. (**a**) Image of the urban sky scene, (**b**) the schematic of the *MASK* matrix of the urban scene, and (**c**–**n**) the fusion results. From (**c**–**h**), the position of the sun $S(\theta_{s},\psi_{s})$ equals $\left(\frac{1}{2}\pi ,0\right)$, $\left(\frac{5}{12}\pi ,0\right)$, $\left(\frac{1}{3}\pi ,0\right)$, $\left(\frac{1}{4}\pi ,0\right)$, $\left(\frac{1}{6}\pi ,0\right)$, and $\left(\frac{1}{12}\pi ,0\right)$. From (**i**–**n**), the position of the sun $S(\theta_{s},\psi_{s})$ equals $\left(\frac{5}{12}\pi ,\frac{1}{6}\pi \right)$, $\left(\frac{5}{12}\pi ,\frac{1}{3}\pi \right)$, $\left(\frac{5}{12}\pi ,\frac{1}{2}\pi \right)$, $\left(\frac{5}{12}\pi ,\frac{2}{3}\pi \right)$, $\left(\frac{5}{12}\pi ,\frac{5}{6}\pi \right)$, and $\left(\frac{5}{12}\pi ,\pi \right)$.

**Figure 8:**
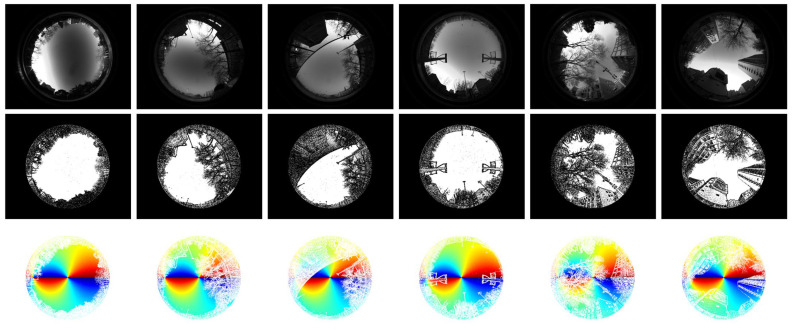
The diversity of landscapes demonstrated by fusion results of different urban scenes when the sun is at the same position. From up to down, the first line shows polarization images captured by the polarimeter under different urban environments. The second line displays the corresponding *MASK* matrixes generated from the images in the first line. The third line displays the fusion results of the above scenes when the position of the sun is at $\left(\frac{5}{12}\pi ,\pi \right)$.

The diversity of the data acquired by the simulator is displayed in Figure 7 and Figure 8. Figure 7 shows some fusion results of the same urban scene when the sun is at different positions. As illustrated in Figure 7, the full-time polarization pattern of a sky scene can be easily acquired by simply changing the coordinate of the sun. Figure 8 shows some fusion results of different urban scenes when the sun is in the same position. As illustrated in Figure 8, no matter the kind of landscape, buildings, trees, poles, or fences, the *MASK* matrix represented the obscurations finely, and the fusion results included abundant scene information.

In summary, as shown in Figure 6, Figure 7 and Figure 8, our simulator provides an effective data acquirement method for data-driven studies in the field of bionic polarization navigation. It can generate a large number of urban skylight polarization pattern data with ground truth based on a few practical measurements, which consumes very little time and labor. The flexible virtual part guarantees the data diversity and abundant landscape information captured by the real part. 

Furthermore, we contracted a skylight polarization pattern dataset based on the experimental results. The dataset provides 174 original polarization images, 174 corresponding MASK matrixes, and full-time skylight polarization patterns under 174 urban scenes, comprising 32,400 urban polarization patterns. The dataset was released at https://pan.baidu.com/s/1raRLVhYk2XqKHIbfiCaPkg?pwd=bitq (accessed on 31 July 2023).

## 4. Conclusions and Prospects

In this paper, we proposed a new virtual-real-fusion framework to acquire data in the field of bionic polarization navigation. We constructed a skylight polarization pattern simulator according to the framework that complements the defects of both methods of practical measurement and numerical simulation. This simulator combines the 3D ideal skylight polarization model with the 2D real urban scene images through calibration and projection, equipping the acquired data with both landscape information and complete ground truths. The experimental results showed that the simulator can simulate the full-time polarization pattern of a specific sky scene, and acquire a large number of urban skylight polarization pattern data with great diversity based on few practical measurements. Based on our experimental results, we released a skylight polarization pattern dataset.

Our work provides researchers with a tool for data preparation and further promote data-driven research on bionic polarization navigation. With more and more machine learning methods applied in the field of bionic polarization navigation [9,10,11,12,13], our dataset can be used for corresponding training or validation. Since our data set is aimed at urban environments, its usage can improve the accuracy of bionic polarization navigation in urban environments. The abundant landscape information can help to overcome the obscuration problems in urban environments and may improve the generalization ability of the network model. Our dataset also provides complete ground truth of the whole skylight polarization pattern, which may benefit studies on pattern inpainting. Moreover, the ground truth in the obscured region may also facilitate the studies on the generation mechanism of polarization patterns on obscurations.

Beyond this paper, our work has more possibilities. We just offer a general framework for the simulator in this paper, but some adjustments are also compatible. In the real part, we constructed an imaging DoFP polarimeter with a fisheye lens to achieve better effects. In fact, other polarimeters, such as the division-of-time polarimeter and the division-of-channel polarimeter, are also appropriate. The type of lens is also adjustable. The imaging model and calibration method used in the fusion part, however, would have to change accordingly. In the virtual part, we used the classic Rayleigh scattering model, but other models, for example, the Berry model and the Hošek–Wilkie model, are also acceptable. Because of the variety of polarization sensors and sky models, to assist researchers to build their customized datasets based on their own polarimeters and sky models, we disclosed the source code of the simulator on GitHub. The code is available at https://github.com/7ianhui/polarization_pattern_data (accessed on 31 July 2023).

Limitations still exist in the skylight polarization pattern simulator proposed in this paper. Because the specific interaction between the skylight and landscapes is very complex and unpredictable, similar to a black box, the current simulator can only fuse the contour information of landscapes into the skylight polarization pattern instead of the precise perturbation introduced by landscapes. This limitation also points out the next research direction. As for solving this “black box” problem and generating the perturbed polarization pattern in those landscape regions, an artificial neural network may help.

## Figures and Tables

**Table 1:** Results of three calibration experiments (unit: pixel).

Group	Internal Parameters				Distortion Coefficient			
	$f_{x}$	$f_{y}$	$c_{x}$	$c_{y}$	$k_{1}$	$k_{2}$	$k_{3}$	$k_{4}$
1	256.44	256.40	607.26	512.57	0.02029	−0.00776	0.00235	−0.00060
2	255.53	255.42	607.61	512.66	0.02211	−0.00908	0.00507	−0.00164
3	256.27	256.19	607.04	513.62	0.02016	−0.00303	0.00093	−0.00092

## Data Availability

Data supporting reported results can be found at https://pan.baidu.com/s/1-BDC8wpDiclBMjGcLspCdw?pwd=ajxa.
